# Comparative study of cerebrospinal fluid α‐synuclein seeding aggregation assays for diagnosis of Parkinson's disease

**DOI:** 10.1002/mds.27646

**Published:** 2019-03-06

**Authors:** Un Jung Kang, Amelia K. Boehme, Graham Fairfoul, Mohammad Shahnawaz, Thong Chi Ma, Samantha J. Hutten, Alison Green, Claudio Soto

**Affiliations:** ^1^ Department of Neurology Columbia University Medical Center New York New York USA; ^2^ The National CJD Research & Surveillance Unit, Western General Hospital University of Edinburgh Edinburgh United Kingdom; ^3^ Mitchell Center for Alzheimer's Disease and Related Brain Disorders University of Texas‐Houston Medical School Houston Texas; ^4^ Michael J Fox Foundation for Parkinson's Research New York New York USA

**Keywords:** Parkinson's disease, α‐synuclein, aggregation, biomarker

## Abstract

**Background:**

PD diagnosis is based primarily on clinical criteria and can be inaccurate. Biological markers, such as α‐synuclein aggregation, that reflect ongoing pathogenic processes may increase diagnosis accuracy and allow disease progression monitoring. Though α‐synuclein aggregation assays have been published, reproducibility, standardization, and validation are key challenges for their development as clinical biomarkers.

**Objective:**

To cross‐validate two α‐synuclein seeding aggregation assays developed to detect pathogenic oligomeric α‐synuclein species in CSF using samples from the same PD patients and healthy controls from the BioFIND cohort.

**Methods:**

CSF samples were tested by two independent laboratories in a blinded fashion. BioFIND features standardized biospecimen collection of clinically typical moderate PD patients and nondisease controls. α‐synuclein aggregation was measured by protein misfolding cyclic amplification (Soto lab) and real‐time quaking‐induced conversion (Green lab). Results were analyzed by an independent statistician.

**Results:**

Measuring 105 PD and 79 healthy control CSF samples, these assays showed 92% concordance. The areas under the curve from receiver operating characteristic curve analysis for the diagnosis of PD versus healthy controls were 0.93 for protein misfolding cyclic amplification, 0.89 for real‐time quaking‐induced conversion, and 0.95 when considering only concordant assay results. Clinical characteristics of false‐positive and ‐negative subjects were not different from true‐negative and ‐positive subjects, respectively.

**Conclusions:**

These α‐synuclein seeding aggregation assays are reliable and reproducible for PD diagnosis. Assay parameters did not correlate with clinical parameters, including disease severity or duration. This assay is highly accurate for PD diagnosis and may impact clinical practice and clinical trials. © 2019 The Authors. *Movement Disorders* published by Wiley Periodicals, Inc. on behalf of International Parkinson and Movement Disorder Society.

Parkinson's disease (PD) diagnosis relies primarily on clinical identification of cardinal motor symptoms (bradykinesia, rigidity, and resting tremor). However, clinical manifestation of PD is heterogeneous and overlaps with other related neurodegenerative disorders, which can hinder accurate diagnosis.^1^, ^2^, ^3^, ^4^, ^5^ Although many studies have reported biochemical differences in biofluids from PD and healthy control (HC) patients, these differences are often at the population level, with significant overlap between groups, and are not useful diagnostic tests for individual patients.^6^ Currently, dopaminergic imaging methods provide the highest accuracy for diagnosing parkinsonism, although they neither distinguish PD from parkinsonism with dopaminergic deficit nor report on the pathogenesis of decreased dopamine levels.^7^ Therefore, reliable biomarkers for common features and pathogenic processes will increase the accuracy of diagnosis, and facilitate clinical care and therapeutic development.

α‐Synuclein dysfunction plays a prominent role in PD pathogenesis. Aggregated α‐synuclein protein is a major constituent of Lewy bodies, and mutations in the *SNCA* gene encoding α‐synuclein cause genetic forms of PD.^8^, ^9^, ^10^ α‐Synuclein protein exists in monomeric and oligomeric forms; in PD, α‐synuclein oligomers adopt conformations that promote aggregation and are thought to confer pathogenicity.^11^, ^12^ These “pathologic” oligomers can act as prions to propagate their conformation to native α‐synuclein.^12^ Recent studies reported that cerebrospinal fluid (CSF) from PD patients can seed aggregation of recombinant α‐synuclein in vitro, which showed a high degree of concordance with PD diagnosis.^13^, ^14^, ^15^ However, the lack of cross‐validation of potential biomarkers is a major challenge in the field to ensure assay validity and reproducibility for further development.^16^ Therefore, we undertook a blinded study of two similar assays that were separately developed by independent laboratories. These two platforms tested CSF from the same subjects in the BioFIND cohort, which utilized standardized biospecimen collection protocols of clinically typical and well‐defined moderate PD patients and healthy controls.^17^, ^18^

## Materials and Methods

### BioFIND study and design

All study protocols were approved by the institutional review board of the individual sites and for the University of Rochester Clinical Trials Coordination Center (CTCC) as outlined in the original BioFIND manuscript.^18^ Moderate‐to‐advanced PD participants with clinically typical features meeting basic and supportive United Kingdom PD Society Brain Bank clinical diagnostic criteria representing all H & Y stages and HC subjects were enrolled at eight movement disorders centers in the United States.^18^ Participants were selected as a convenience series for those who volunteered for the biomarker study without randomization or consecutive enrollment. Additional inclusion criteria for PD patients included: expression of all three classic parkinsonian motor signs (bradykinesia, rigidity, and resting tremor) by history or examination; disease duration ≥5 years and onset between ages 50 and 75 years; and well‐established response to dopaminergic agents and/or amantadine. Subjects were excluded if they had: features of atypical or secondary parkinsonian syndromes; history of DBS or ablative brain surgery; history of cancer (except basal or squamous cell skin cancers) within 5 years preceding enrollment; autoimmune, liver, or hematological disorders; or conditions precluding lumbar puncture. HCs were matched by sex and age to PD subjects, were free of any known neurological disorders, and scored ≥26 on the Montreal Cognitive Assessment (MoCA). Other exclusion criteria for HCs were similar to those for PD subjects. Additionally, HC subjects with any first‐degree family member with PD were excluded to reduce the chance of enrolling prodromal cases. Additional details regarding the cohort are discussed in the original manuscript detailing the BioFIND study.^18^

### Evaluations

Clinical evaluation and biospecimen collection occurred during two visits, one to establish the baseline (V1) and a follow‐up 2 weeks later (V2). For PD subjects, V1 was performed during the *on* state (1–3 hours after the last PD medication dose) and V2 was performed in the practically defined *off* state (early morning before PD medications and approximately 12 hours after the last dose the night before). During V1, blood for DNA and plasma were collected and clinical assessments were made including the International Parkinson and Movement Disorder Society–sponsored revision of the UPDRS (MDS‐UPDRS) Parts I (nonmotor experiences of daily living), II (motor experiences of daily living), III (motor examination), and IV (motor complications) for PD subjects and only Part III for HCs. During V2, blood for RNA and plasma, CSF, and saliva (in a smaller subgroup) were collected, and the MDS‐UPDRS Part III was administered to PD subjects. All subjects either fasted or consumed a low‐fat diet on the morning of V2 at the time of biospecimen collection. CSF was immediately frozen in a –80^°^C freezer and shipped to the biorepository on dry ice at a later date.^18^ Data collected included demographics, family history of PD, medical/neurological histories, medications, neurological exams, the MDS‐UPDRS,^19^ MoCA,^20^ and REM Behavior Sleep Disorder (RBD) Questionnaire^21^ and, for PD subjects, the Modified Schwab and England Activities of Daily Living Scale.^22^

### Synuclein Seeding Aggregation Assays

Two independent laboratories performed their respective assays on separate aliquots of CSF from the same subjects. Both assays are based on the ability of pathological α‐synuclein oligomers to seed aggregation at the expense of exogenously supplied monomeric α‐synuclein protein. Cyclic amplification of protein misfolding (PMCA) adapted for α‐synuclein oligomer formation was performed by the Soto group,^14^ and the real‐time quaking‐induced conversion (RT‐QuIC) assay was performed by the Green group.^15^ Both assays are recent adaptations of the original PMCA method, first developed for misfolded prion proteins by the Soto lab^23^ and streamlined to use fluorescence detection by the lab of Byron Caughey.^24^ Differences include pH, shaking conditions, and source of the recombinant α‐synuclein protein as summarized and compared to a recent publication of an α‐synuclein seeding aggregation assay (SAA) by the Caughey lab^13^ in Supporting Information Table S1.

#### RT‐QuIC

The RT‐QuIC assay was performed as previously described.^15^ The reaction buffer (RB) was composed of 100 mM of phosphate buffer (pH 8.2), 10 μM of Thioflavin T (ThT), and 0.1 mg/mL of human recombinant full‐length (1–140 amino acids) α‐synuclein (Lot No: 056M4113V; Sigma‐Aldrich Ltd, Poole, UK). Each well of a black 96‐well clear bottom plate (Nalgene Nunc International, Fisher Scientific Ltd, Loughborough, UK) contained 98, 90, or 85 μL of RB (depending on volume of seed added) and 37 ± 3 mg of 0.5‐mm zirconium/silica beads (Thistle Scientific Ltd, Glasgow, UK). Reactions were seeded with 2 μL of working‐strength brain homogenate or 15 μL of undiluted CSF to a final reaction volume of 100 μL. Plates were sealed with a plate sealer film (Fisher Scientific Ltd, Loughborough, UK) and incubated in a BMG Labtech FLUOStar OPTIMA plate reader at 30°C for 120 hours with intermittent shaking cycles: double orbital with 1‐minute shake (200 rpm), 14‐minute rest. ThT fluorescence measurements (450 nm excitation and 480 nm emission) were taken every 15 minutes. Each sample was run in duplicate. A positive response was defined as a relative fluorescence unit (RFU) value of >2 standard deviations above the mean of the negative controls at 120 hours in both of the CSF duplicates. If only one of two CSF sample replicates gave a positive response, the RT‐QuIC analysis of the CSF samples was repeated in quadruplicate. A positive response in two or more replicates was considered positive, otherwise the sample was considered negative. Twenty four samples were repeated because of discordant findings between technical duplicates on the first run. Seventeen of these showed concordance with clinical diagnosis on repeats. Five were false positives and two were false negatives. The final fluorescence value was the mean fluorescence value taken at 120 hours. The maximum fluorescence value was the highest mean fluorescence value recorded during the RT‐QuIC analytical run of 120 hours. The latency to reach 50% of the maximal fluorescence value was noted as T50. Examples of ThT curves are shown in Supporting Information Figure S1. Intrasample precision was 12.1%. This was calculated from repeated analysis of individual aliquots of a CSF sample from a PD patient over the 12‐month study period.

#### PMCA

The PMCA assay was performed as previously described.^14^ Briefly, recombinant full‐length α‐synuclein (containing a his‐tag for purification) at a concentration of 1 mg/mL in 100 mM of PIPES (pH 6.5), 500 mM of NaCl, were placed in opaque 96‐well plates in the presence of 5 μM of ThT at a final volume of 200 μL. For each test, we added 40 μL of CSF from patients and controls. Positive controls consisted of a well‐documented and previously screened healthy CSF sample spiked with in vitro generated α‐synuclein oligomeric seeds. Samples were subjected to cyclic agitation (1 minute at 500 rpm followed by 29 minutes without shaking) at 37°C for 400 hours. The increase in ThT fluorescence was monitored at excitation of 435 nm and emission of 485 nm, periodically, using a microplate spectrofluorometer (Gemini‐EM; Molecular Devices, Sunnywale, CA). We set an experimentally defined threshold of 1,000 fluorescence units as the cut‐off criteria for a “positive” designation for each sample (samples below 1,000 were classified as “negative”). Each sample was assayed in triplicate, which showed a high degree of concordance. One sample was defined as uncertain positive, because the maximum fluorescence was higher than 1,000 in only two of the three replicates. Three samples were defined as uncertain negative, because two of the three replicates did not show aggregation. These results were concordant with the clinical diagnosis and RT‐QuIC results.

The PMCA assays for α‐synuclein showed specificity toward α‐synuclein seed compared to β‐amyloid (1‐42) and tau proteins.^14^ In addition, immunodepletion of α‐synuclein from the CSF significantly delayed time needed to reach 50% of maximum fluorescence (T_50_), indicating removal of the majority of the seeds. When the same samples were used for assay at a later time and when measured after one versus two freeze‐thaw cycles, a good reproducibility of data was demonstrated previously. Varying seed concentrations resulted in corresponding changes in T_50_.^14^ Dilution curve of BioFIND reference pool CSF also showed corresponding changes in T_50_ (Soto, unpublished).

### Statistical Analysis

Differences between cases and controls were compared using percent and frequency for categorical data and median and range for continuous data. Categorical data were assessed using chi‐square or Fisher's exact test, where appropriate, and differences in continuous data was assessed using Wilcoxon rank sum. Sensitivity, specificity, negative predictive value (NPV), and positive predictive value (PPV) were calculated for comparisons between biomarker results and clinical diagnosis. Correlations were calculated for biomarker results between the two techniques as well as between biomarker results and clinical characteristics. Logistic regression was used to generate receiver operating characteristic (ROC) curves to assess the relationship between biomarker results and clinical diagnosis of PD. A two‐tailed alpha of 0.05 was used.

### Data Availability Statement

Individual de‐identified participant data consisting of clinical phenotypes, demographics, and assay data are available online (http://biofind.loni.usc.edu/). The data are publically available upon registration by the Michael J. Fox Foundation for Parkinson's Research. The study protocol is available (https://www.michaeljfox.org/page.html?biofind‐clinical‐study). The statistical analysis is available upon request from the authors.

## Results

**Figure 1:**
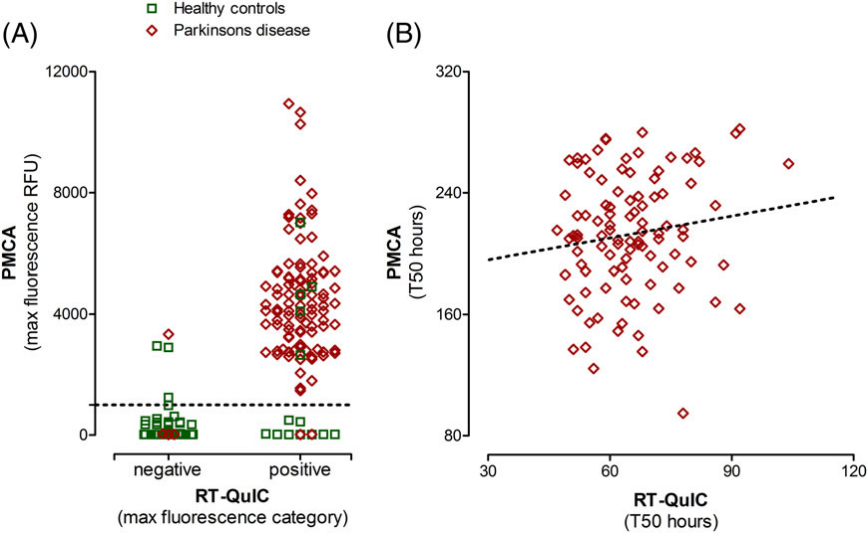
Distribution of assay parameters in BioFIND subjects. Maximum fluorescence values were used to designate positive or negative aggregation results. The PMCA assay yielded graded maximum fluorescence whereas the RT‐QuIC values were binary. (A) Maximum fluorescence values from the PMCA assay were plotted against positive or negative designations in the RT‐QuIC assay. The dotted line designates the cut‐off threshold for a positive result by PMCA (1,000 RFUs). (B) T_50_ of the PMCA assay was plotted against the RT‐QuIC assay. Green boxes = healthy control subjects; red diamonds = PD subjects; dotted line = linear regression line (R^2^ = 0.47; *P* < 0.0001).

A total of 105 PD subjects and 79 HC subjects with results from both assays were included in the analysis. Subject demographics are shown in Table 1. Whereas both assays were similar (Supporting Information Table S1), the PMCA assay gave a range of maximum fluorescence values, whereas the RT‐QuIC assay yielded either positive or negative signal based on the endpoint fluorescence level (Fig. 1A). Overall, 92% of subjects showed concordant results across both assays. Eleven subjects were positive by RT‐QuIC, but negative by PMCA, and 4 subjects were positive by PMCA, but negative by RT‐QuIC (Fig. 1A). Time needed to reach T_50_ varied among the positive samples in both assays, and all the samples with negative results based on the maximum fluorescence had no measurable T_50_ values. Though the maximum fluorescence and T_50_ values for the PMCA assay were continuous, they showed only moderate correlation (R^2^ = 0.5569 for PMCA; *P* < 0.0001). T_50_ values in PD subjects from two assays were only weakly correlated (R^2^ = 0.4748; *P* < 0.0001; Fig. 1B).

**Table 1:** Demographic characteristics in the BIOFIND samples

	Total (N = 184)	Cases (N = 105)	Controls (N = 79)	
	N (%)	N (%)	N (%)	*P* Value
Male, %	101 (54.9)	63 (62.4)	38 (37.6)	0.108
White, %	171 (92.3)	99 (94.3)	72 (91.1)	0.410
	Median (range)	Median (range)	Median (range)	
Age at enrollment	66 (55–84)	68 (55–82)	65 (53–84)	0.997
MoCA	28 (19–30)	27 (19–30)	28 (26–30)	0.0003
RBD score	3 (0–13)	5 (0–13)	2 (0–8)	0.0001
RBD >5, %	50 (27.2%)	47 (44.8%)	3 (3.8%)	0.0001
TD, %	—	51.4%	—	—
PIGD, %	—	37.1%	—	—
α‐Synuclein	1,469.5 (378–3,852.8)	1,421.4 (378–3,449.3)	1,590.7 (86.3–3,852.8)	0.009
β‐Amyloid (1‐42)	323.1 (11.4–500.7)	289 (123.6–468.3)	354.9 (11.4–500.7)	0.003
t‐tau	35.3 (1.0–172.4)	33.6 (1.0–98.9)	36.1 (1.0–172.4)	0.115
p‐tau	13.9 (4.3–127.9)	12.6 (4.3–76.6)	14.6 (5.4–127.9)	0.0542
Age at PD onset	—	61 (51–76)	—	—
Duration of PD since onset	—	8 (4–17)	—	—
LEDD	—	680 (100–2,045.5)	—	—
UPDRS part I	—	9 (0–27)	—	—
UPDRS part II	—	11 (0–34)	—	—
UPDRS part III *off*	12.5 (0–66)	26 (7–66)	1 (0–10)	0.0001
UPDRS part IV	—	4 (0–12)	—	—
UPDRS total *on*	—	46 (12–127)	—	—
UPDRS total *off*	—	59 (22–133)	—	—
H & Y stage	—	2 (1–4)	—	—

Abbreviations: TD, tremor‐dominant subtype; PIGD, postural instability and gait disorders subtype; LEDD, levodopa‐equivalent daily dose.

Overall, both assays exhibited high accuracy for clinical diagnosis of PD. Areas under the curve (AUCs) of ROC curves were 0.94 when considering only the samples with concordant results between both assays. Separately, AUCs for the PMCA and RT‐QuIC assays were 0.93 and 0.89, respectively (Table 2). Sensitivity, specificity, PPV, and NPV and AUC from ROC analysis are shown in Table 2.

**Table 2:** Predictability of assays for PD diagnosis

	Only Assay Concordant Subjects Included	PMCA	RT‐QuIC
Sensitivity	97.1% (92.9–99.1)	95.2% (90.6–98.0)	96.2% (91.4–98.7)
Specificity	92.5% (86.2–95.7)	89.9% (83.8–93.5)	82.3% (76.0–85.6)
PPV	95.2% (91.1–97.2)	92.6% (88.1–95.2)	87.8% (83.5–90.1)
NPV	95.4% (88.9–98.6)	93.4% (87.1–97.2)	94.2% (87.0–98.0)
AUC	0.9480	0.9256	0.8923

Sensitivity, specificity, PPV, NPV, and AUC of the ROC analysis. Values in parentheses indicate 95% confidence intervals. Of 105 PD and 79 HC subjects, the assay results were concordant in 102 PD and 67 HC subjects.

**Figure 2:**
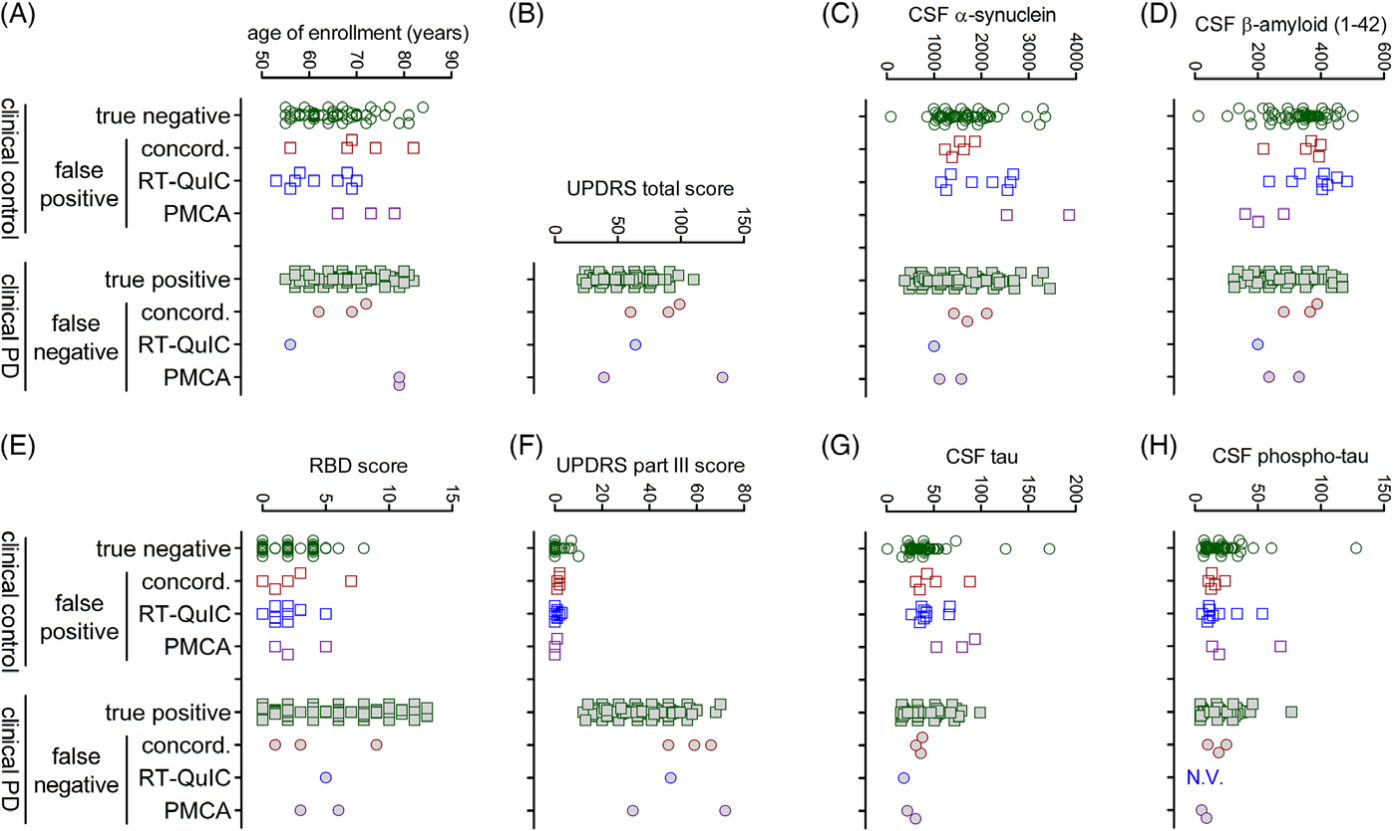
Comparison of clinical characteristics and CSF analyte values of subjects with assay results discordant with clinical diagnosis. Values from false‐positive and ‐negative α‐synuclein seeding assay results common to both assays, or unique to each assay, were compared to the true‐negative and ‐positive subjects, respectively. There were no differences for (A) age of enrollment, (B) UPDRS total score, (C) CSF α‐synuclein levels, (D) CSF β‐amyloid (1‐42) levels, (E) RBD score, (F) UPDRS part III score, (G) CSF tau levels, and (H) phospho‐tau protein levels.

**Figure 3:**
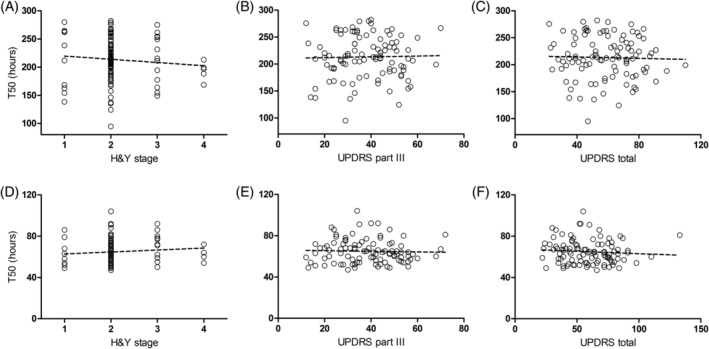
Correlation of T_50_ values to disease characteristics for PD patients. T_50_ values of the PMCA (A–C) and RT‐QuIC (D–F) assays were plotted against (A and D) H & Y stage, (B and E) total UPDRS part III, and (C and F) UPDRS total scores for PD patients with positive assay results. For the PMCA assay: n = 100, (A) R^2^ = 0.0099, *P* = 0.3235, (B) R^2^ = 0.0013, *P* = 0.7202, and (C) R^2^ = 0.0004, *P* = 0.8458. For the RT‐QuIC assay: n = 101, (D) R^2^ = 0.0093, *P* = 0.3365, (E) R^2^ = 0.0039, *P* = 0.5338, and (F) R^2^ = 0.0100, *P* = 0.3204.

Clinical characteristics of false‐positive and ‐negative subjects, including age at enrollment, UPDRS total and UPDRS part III scores, H & Y stage, and RBD score were not significantly different from true‐negative and ‐positive subjects, respectively (Fig. 2). This was also the case for CSF α‐synuclein, β‐amyloid (1‐42), total tau (t‐tau), and tau phosphorylated at threonine 181 (p‐tau) levels (Fig. 2). Although predictive values of the SAAs were high for the diagnosis of PD, the primary assay parameters of maximum fluorescence and T_50_ did not correlate with any clinical parameter, including disease duration, age, sex, or UPDRS scores (Supporting Information Table S2; Fig. 3). We also tested whether maximum fluorescence or T_50_ values from the α‐synuclein SAAs correlated with total CSF α‐synuclein, β‐amyloid, t‐tau, and p‐tau levels and did not see any significant relationships (Supporting Information Table S2).

Though the BioFIND cohort was purposely designed to reduce the chances of including genetic forms of PD, common glucocerebrosidase (GBA) and leucine repeat‐rich kinase 2 (LRRK2) variants were present in a few PD patients and HCs without PD.^25^ Based on published studies, we hypothesized that GBA mutations may increase α‐synuclein pathology whereas some LRRK2 mutations may lack α‐synuclein pathology.^26^, ^27^ We had 7 PD and 2 HC subjects with pathogenic GBA variants, and 1 PD and 1 HC subject with the LRRK2 G2019S mutation. However, the assay results from these genetic variants of PD did not show any differences from subjects without established abnormal genetic variants and were concordant with their clinical diagnosis.

## Discussion

Development of new biomarkers assays often face significant challenges in replicability in additional patient cohorts. Because of the complexity of obtaining and assaying human samples, variability may arise in many areas, including from differences in patient enrollment, biospecimen collection and processing, and assay execution. Thus, independent replication and cross‐validation of assays is a critical step that is often lacking in the literature. Therefore, we utilized the BioFIND cohort, a standardized benchmark cohort of PD patients, for blinded assay validation of two α‐synuclein aggregation assays based on the sample principle. These assays use CSF to seed the aggregation of monomeric α‐synuclein protein and are among the most promising biomarkers for PD diagnosis. Our results validate the preliminary studies and show robust power to confirm PD diagnosis from the same CSF samples at the individual level.^13^, ^14^, ^15^ This is contrasted by the consistent finding that total α‐synuclein protein levels are lower in CSF from PD patients compared to HCs at the population level, with significant overlap between the two groups.^17^, ^28^, ^29^

Although the concordance rate was high between the two assays (92%; Fig. 1), we did not find any systematic explanation for the discrepant findings. This included comparison of the borderline or nonunanimous results between assays, clinical and biochemical characteristics of patients, and correlation of assay parameters (i.e., samples with longer T_50_ times in one assay lacking aggregation in the other). Thus, discordant results may be attributed to the slight differences in the conditions, reagents, and procedures for each assay (Supporting Information Table S1) and may improve with further optimization.

In both assays, maximum fluorescence values were used to designate a “positive” result. The RT‐QuIC assay was optimized for maximum sensitivity for PD detection, yielding nearly all‐or‐none fluorescence levels.^15^ The PMCA assay produced a range of maximum fluorescence values and used an experimentally derived cut‐off value (1,000 RFUs in this study) for characterization (Fig. 1A). These differences resulted in higher sensitivity of RT‐QuIC (96.2% vs. 95.2%) and higher specificity of PMCA (89.9% vs. 82.3%; Table 2). When only considering samples with concordant results from both assays, specificity of the assay improved to 92.5% and sensitivity increased to 97.1%, suggesting that using both assays together would further improve diagnostic performance. These α‐synuclein SAAs have the highest diagnostic accuracy for PD biomarkers reported in the literature. Head‐to‐head comparison with DATSCAN in the same cohort will be useful in determining whether α‐synuclein SAAs can be substituted for expensive imaging for PD diagnosis.

In this study, both assays yielded a varying range of T_50_ values (Fig. 1B), suggesting that this parameter may reflect the amount of α‐synuclein seeding species present in the assay. In a previous study, T_50_ values of the PMCA assay correlated to the amount of α‐synuclein oligomer seed spiked into control CSF and the H & Y stage in patient CFS samples.^14^ However, T_50_ values from either assay did not correlate to any clinical parameter in the present study (Supporting Information Table S2; Fig. 3). Given that the T_50_ values showed only weak correlation between assays, the current assay conditions may not reflect quantitative aspects of the seeding capacity of CSF samples (Fig. 1B), which may also be true for the maximum fluorescence values. A similar assay published recently^13^ used nondisease K23Q mutant α‐synuclein protein (Supporting Information Table S1) with a shortened assay time and demonstrated high sensitivity and specificity, though only a small number of CSF samples were assayed.^13^ Whether our results indicate that the level of pathogenic α‐synuclein species capable of seeding aggregation in the CSF do not change with disease stage or reflect limitations of the assays as implemented are currently being addressed. The BioFIND cohort contains patients with H & Y stage 1 to 4 with a mean of 2.1 and lacks de novo and H & Y stage 5 patients. Further studies with a wider spectrum of PD stages, including prodromal stages, and a longitudinal cohort will be important to address whether levels of pathological forms of α‐synuclein in CSF are changed with disease severity and progression.

Importantly, these assays probe a process that may be central to PD pathogenesis and strongly support that misfolded α‐synuclein protein present in CSF of PD patients can seed the aggregation of monomeric α‐synuclein protein by propagating an aberrant protein conformation in a prion‐like fashion. Although data collection from the BioFIND cohort ended with the final visit, precluding the possibility of obtaining pathology information, a previous study using the RT‐QuIC assay included 2 PD, 11 dementia with Lewy bodies, 30 Alzheimer's disease, and 20 HC subjects with pathological confirmation of diagnosis and showed high sensitivity and specificity for Lewy body diseases.^15^ Though CSF from atypical parkinsonism cases also seed aggregation in these assays,^13^, ^14^, ^15^ the physical nature of these aggregates may differ based on the disease. For instance, aggregates induced by α‐synuclein obtained from glial cytoplasmic inclusions in MSA have different properties than those seeded from Lewy body (LB)‐derived α‐synuclein.^30^ We are currently developing methods to identify the types of aggregates formed in the SAAs, which may be useful to differentiate PD from atypical parkinsonism. Importantly, CSF from tauopathies, such as supranuclear palsy, did not seed α‐synuclein aggregation in either assay.^14^, ^15^ Of note, there are rare cases of PD that lack LB pathology, including the R1441 and G2019S LRRK2 mutations, in which the role of synucleinopathy in disease pathogenesis is questioned.^26^, ^27^, ^31^ There were 2 cases of G2019S LRRK2 mutation in the present BioFIND cohort: 1 PD and HC subject. Both α‐synuclein SAAs showed results concordant with the clinical diagnoses.

Though CSF samples were obtained after overnight withdrawal of medication, all PD patients in the BioFIND cohort were treated with dopaminergic drugs and/or amantadine for symptomatic control. Although these drugs have been shown not to affect total α‐synuclein levels in plasma and CSF,^32^, ^33^ we cannot rule out the effect of dopaminergic medication on α‐synuclein aggregation. The previous studies using these SAAs did not specify medication use in their PD subjects.^13^, ^14^, ^15^ Inclusion of de novo PD subjects, who are drug naïve, such as those included in the PPMI cohort, would address the effect of dopaminergic drugs on seeding ability of CSF α‐synuclein.

Overall, our results show robust power of α‐synuclein SAAs to predict clinical diagnosis in a typical moderate PD cohort by independently cross‐validating two different assay platforms using the same CSF samples. Given that these assays report on a process central to disease pathogenesis, they have the potential to be a surrogate for the presence of α‐synuclein pathology and should be tested with larger cohorts of patients with pathological confirmation of PD compared to other pathologies. Such predictive power will increase the rigor for PD diagnosis and improve subject selection for therapeutic trials. Additionally, these assays may provide the opportunity to directly evaluate target engagement and efficacy of therapeutic modalities that target α‐synuclein levels or aggregation. Whether SAAs can aid in earlier diagnosis by detecting pathogenic processes during prodromal phases before clinical manifestation remains to be systematically studied. CSF from the same 5 HC subjects was classified positive by both assays. Although informal follow‐up of 3 of these patients did not show conversion to a PD phenotype, long‐term follow‐up is necessary to determine whether these “false positives” were actually prodromal cases. Likewise, whether these assays have utility in tracking disease progression and severity will require further development. In addition, the utility of these assays needs to be further evaluated in a wider range of cross‐sectional and longitudinal cohorts.

Given the high concordance of the results obtained by the PMCA and RT‐QuIC assays, the similarity on the principles and methodology, it would be advisable to unify the name of these assays to avoid confusion in the literature. We propose to use the term “seeding aggregation assay (SAA)” to refer to these tests in order to highlight the basic principle behind them. Based on their high predictive power, these α‐synuclein SAAs tested in our study have the potential to revolutionize PD diagnosis for clinical practice and clinical trials.

## Author Roles

(1) Research Project: A. Conception and Design; B. Acquisition of Data; C. Analysis and Interpretation of Data; (2) Manuscript: A. Writing of the First Draft, B. Review and Critique; (3) Other: A. Statistical Analysis; B. Obtaining Funding; C. Technical Support; D. Supervision of Data Collection.

U.J.K.: 1A, 1C, 2A, 2B, 3B

A.K.B.: 1C, 2B, 3A

G.F.: 1B

M.S.: 1B

T.C.M.: 1C, 2B

S.J.H.: 1C, 2B, 3B

A.G.: 1A, 1C, 2B, 3B, 3D

C.S.: 1A, 1C, 2B, 3B, 3D

## Financial Disclosures

Nothing to report.

## Supporting information

Figure S1: Supporting information

Supplementary Table 1. Comparison of PMCA and RT‐QuIC methodologies
Supplementary Table 2. Correlation of assay results with clinical parameters and CSF analytes.
