# Supplementary figures and images for: Comparative study of cerebrospinal fluid α‐synuclein seeding aggregation assays for diagnosis of Parkinson's disease
Source: Mov Disord. 2019 Mar 6;34(4):536–44. doi: 10.1002/mds.27646 (PMC6519150; doi:10.1002/mds.27646)

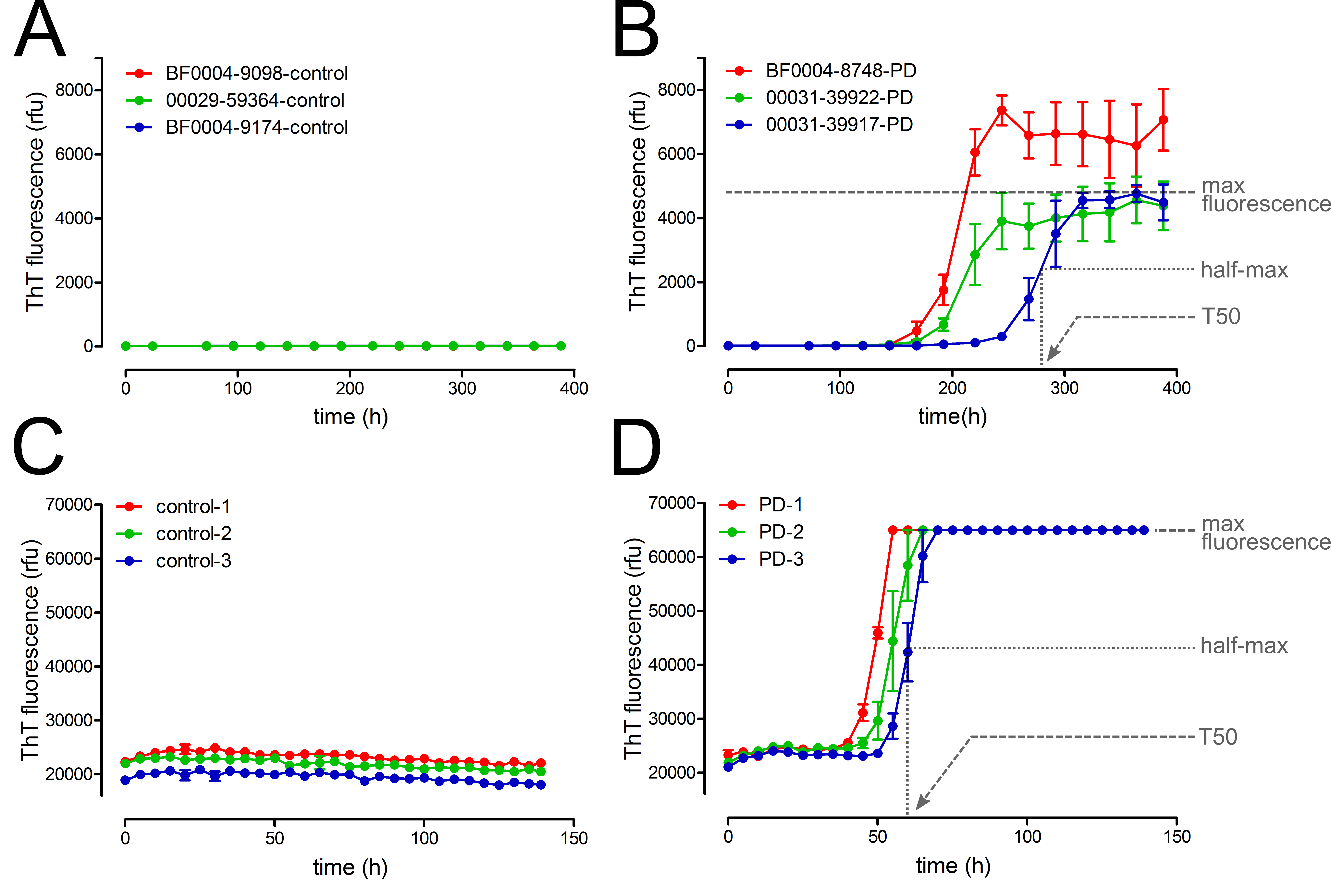

Supplement: Supplementary file 1 — Figure S1: Supporting information [file MDS-34-536-s001.tif]
