# Supplementary material for: Comparative study of cerebrospinal fluid α‐synuclein seeding aggregation assays for diagnosis of Parkinson's disease
Source: Mov Disord. 2019 Mar 6;34(4):536–44. doi: 10.1002/mds.27646 (PMC6519150; doi:10.1002/mds.27646)
Supplement: Supplementary file 2 — Supplementary Table 1. Comparison of PMCA and RT‐QuIC methodologies Supplementary Table 2. Correlation of assay results with clinical parameters and CSF analytes. [file MDS-34-536-s002.docx]

**Comparative study of CSF α-synuclein seeding aggregation assays for diagnosis of Parkinson’s disease**

Un Jung Kang, MD^1^*, Amelia K. Boehme, PhD^1^, Graham Fairfoul, BS^2^**, Mohammad Shahnawaz, PhD^3^**, Thong Chi Ma, PhD^1^, Samantha J. Hutten, PhD^4^, Alison Green, PhD^2^*, Claudio Soto, PhD^3^*

*, ** These authors contributed equally to the manuscript

Supplementary Table 1. Comparison of PMCA and RT-QuIC methodologies

|  | **Substrate** | **Buffer** | **CSF**  **vol.** | **Shaking conditions** | **Criteria for Positive results** |
| --- | --- | --- | --- | --- | --- |
| Soto Lab (2017)^14^ | Human recombinant α-synuclein (in-house)  1 mg/mL | 100 mM PIPES,  500 mM NaCl,  5 µM ThT,  pH 6.5 | 40 µL | 500 rpm  1 min shaking  29 min rest for 400 hr at  37°C | RFU >1000  positive if 3/3  uncertain if 2/3 |
| Green Lab (2016)^15^ | Human recombinant α-synuclein (1-140 aa)  (Sigma, lot #: 56M4113V)  0.1 mg/mL | 100 mM phosphate buffer,  10 µM ThT,  pH 8.2 | 15 µL | 200 rpm  1 min shaking  14 min rest for 120 hr at  30°C | RFU > 2SD of avg  positive if 2/2  if 1/2 🡪 repeat with 4 replicates positive if ≥ 2/4 |
| Caughey  Lab (2018)^13^ | K23Q α-synuclein  (in-house)  0.1 mg/ml | 40 mM phosphate buffer, 0.0015% SDS  10 µM ThT,  pH 8.0 | 15 µL | 400 rpm  1 min shaking  1 min rest for 48 hr at  42°C | RFU > 3SD of avg  positive if ≥ 2/4 |

RFU: relative fluorescence unit

Supplementary Table 2. Correlation of assay results with clinical parameters and CSF analytes.

|  | PMCA | | | RT-QuIC | | |
| --- | --- | --- | --- | --- | --- | --- |
|  | N | R2 | p-value | N | R2 | p-value |
| T50*age | 100 | 0.0283 | 0.0942 | 101 | 0.0032 | 0.5758 |
| T50*sex | 100 | 0.0349 | 0.0627 | 101 | 0.0411 | 0.0420 |
| T50*disease duration | 100 | 0.0037 | 0.5470 | 101 | 0.0189 | 0.1700 |
| T50*UPDRS 3 | 100 | 0.0013 | 0.7202 | 101 | 0.0039 | 0.5338 |
| T50*UPDRS total | 100 | 0.0004 | 0.8458 | 101 | 0.0100 | 0.3204 |
| T50*H&Y stage | 100 | 0.0099 | 0.3235 | 101 | 0.0093 | 0.3365 |
| T50*MoCA | 100 | 0.1073 | 0.0009 | 101 | 0.0006 | 0.8106 |
| T50*α-synuclein | 92 | 0.0070 | 0.4292 | 93 | 0.0073 | 0.4162 |
| T50*β-amyloid (1-42) | 100 | 0.0076 | 0.3874 | 101 | 0.0112 | 0.2921 |
| T50*t tau | 98 | 0.0033 | 0.5728 | 99 | 0.0092 | 0.3437 |
| T50*p-tau | 99 | 0.0339 | 0.0681 | 101 | 0.0248 | 0.1159 |
| Max Fluor*age | 105 | -0.0553 | 0.5754 | 105 | -0.0788 | 0.4243 |
| Max Fluor*sex | 105 | 0.0811 | 0.4108 | 105 | -0.1400 | 0.1542 |
| Max Fluor*disease duration | 105 | 0.0761 | 0.4401 | 105 | 0.1503 | 0.1258 |
| Max Fluor*UPDRS 3 | 105 | -0.1160 | 0.2385 | 105 | -0.1160 | 0.2385 |
| Max Fluor*UPDRS total | 105 | -0.1099 | 0.2642 | 105 | -0.1948 | 0.0465 |
| Max Fluor*H&Y stage | 105 | -0.1445 | 0.1413 | 105 | -0.1369 | 0.1636 |
| Max Fluor*MoCA | 105 | -0.0915 | 0.3532 | 105 | -0.1046 | 0.2882 |
| Max Fluor*α-synuclein | 97 | 0.1246 | 0.2241 | 97 | -0.0730 | 0.4772 |
| Max Fluor*β-amyloid (1-42) | 105 | 0.0697 | 0.4802 | 105 | -0.1054 | 0.2848 |
| Max Fluor*t-tau | 103 | 0.1928 | 0.0510 | 103 | 0.0232 | 0.8160 |
| Max Fluor*p-tau | 104 | 0.0787 | 0.4270 | 104 | -0.0235 | 0.8124 |
